# Answer – Preterm Preeclampsia and Timing of Delivery: A Systematic Literature Review

**DOI:** 10.1055/s-0038-1668530

**Published:** 2018-08

**Authors:** Maria Laura Costa, Jose Paulo de Siqueira Guida, Fernanda Garanhani Surita, Mary Angela Parpinelli

**Affiliations:** 1 Department of Gynecology and Obstetrics, Universidade Estadual de Campinas, Campinas, SP, Brazil

Dear Editor,

We thank Leite T. and Paravidino V. B. for the interest and thoughtful comments, and we agree that the topic of this article is of great clinical relevance.^1^ We acknowledge the concern about methodological issues, such as time period and search strategy; and we hope to further clarify the approach used. The review was supported by the National Brazilian Specialized Committee on Preeclampsia of the Brazilian Federation of Gynecology and Obstetrics Associations (FEBRASGO, in the Portuguese acronym), aiming to enable national awareness regarding the most important cause of maternal mortality and morbidity in our scenario. This group of specialists revised the presented results and sought to ensure a simple and clear text and method, mostly for an audience of clinicians.

The decision of considering the restricted period (between 2014 and 2017) was mainly due to two reasons. The first one was to reflect the new recommendations adopted by the International Society for the Study of Hypertension in Pregnancy (ISSHP), which has broadened the definition of preeclampsia after 2013.^2^ Since then, preeclampsia is diagnosed not only if there is a new onset of hypertension and proteinuria, but also if hypertension and significant end-organ dysfunction without proteinuria occur after 20 weeks of gestation. The second reason was to consider a period after which there was a similar Cochrane review.^3^ A systematic review is a method to synthesize the available evidence using an explicit, transparent approach, and this was indeed performed.

The present review aimed to update the available evidence on the best timing of delivery for preterm preeclampsia. We do understand all the requirements on the Cochrane Handbook for Systematic Reviews and also acknowledge previous published reviews on the topic by the Cochrane initiative.^3^ ^4^ However, the 2013 Cochrane review^3^ considered preeclampsia cases between 24 and 34 weeks of gestation, and the 2017 Cochrane review considered cases between 34 weeks of gestation and term pregnancy.^4^ We have decided to consider both, before and after 34 weeks of gestation, and to present results in a comprehensive way, to guide counseling. This is why we even included a box that presented “How to talk with pregnant mothers and their families about the risks, benefits and uncertainties of immediate delivery versus expectant management when preterm preeclampsia is diagnosed.”

The other key concern about the search strategy is also very relevant. We did initially use many other Medical Subject Headings (MeSh) terms, but chose the simplest combination of terms, with no loss of retrieved articles. To make sure this was true, we have now performed again the same search using the suggested terms and have retrieved the exact same final papers. The same happened with the databases. We should have stated that Lilacs and Embase were searched, but we again chose to present the most straightforward approach.

It is clear from the thoughtful comments presented that there are still unanswered questions on this topic, and we hope to stimulate future studies to guarantee adequate patient care and counseling in cases of preterm preeclampsia. We invite the comment authors to join forces in future researches and reviews on the topic.
